# Tuto, Cito Et Jucunde

**Published:** 1888-03

**Authors:** 


### "TUTO, CITO ET JUCUNDE."

What would one think of a soldier going to fight without being armed and equipped?

What would one think of a surgeon who, being suddenly called to a scene of disaster or accident, would start without his surgical case?

What *should* one think of a physician who, being hurriedly summoned to the chamber of sickness and impending death, would repair thereto without any of those means which observation, experience, not to say education and common sense, should have taught him to be constantly provided with, and to have at all times in his pocket case with a view if not to cure, at least to relieve?

Relief is what nine-tenths of patients stand, or rather lie, in need of!

We can conceive of nothing more pitiable and ridiculous than the condition of the cross armed or thumb revolving physician who is unable to alleviate pain and suffering within fifteen minutes of his arrival without being obliged to write a prescription to be sent sometimes to a considerable distance and not infrequently to be deferred till the primary means to obtain it have been secured!

We will not suggest any remedies, or rather any method, and call it "*My Pocket Case!*" Such a proceeding would imply an imputation and savor of a *panparonade*. Is there a physician reading these lines who does not remember many a case he would have relieved, not to say saved, had he been duly equipped?

Moral: "Remember and Reform!" G.

